# What do service providers in Southern Ethiopia say about barriers to using youth-friendly sexual and reproductive health services for adolescents?: Qualitative study

**DOI:** 10.1186/s12978-021-01092-0

**Published:** 2021-02-09

**Authors:** Yitagesu Habtu, Mirgissa Kaba, Hussein Mekonnen

**Affiliations:** 1 School of Public Health, College of Health Sciences, Addis Ababa University, Addis Ababa, Ethiopia; 2 School of Public Health, College of Health Sciences, Addis Ababa University, Addis Ababa, Ethiopia; 3 School of Nursing, College of Health Sciences, Addis Ababa University, Addis Ababa, Ethiopia

**Keywords:** Barriers, Providers’ perspective, Adolescent, Friendly-sexual and reproductive health service

## Abstract

**Background:**

In Ethiopia, the utilization coverage of adolescent-friendly health services (AFSRHs) ranged only from 9 to 55% and it was the lowest of all Sub-Saharan African countries in 2016. Little is known why adolescents were not accessing the existing services to the side of healthcare providers.

**Objective:**

The aim of this study is to explore contextual perceived and actual barriers to accessing AFSRHs by adolescents in Southern Ethiopia.

**Methods:**

Phenomenological study design supplemented with observation was used to explore perceived and actual barriers to accessing AFSRHs in 2020. Criterion sampling was used to select study participants. In-depth interviews with healthcare providers and non-specialist sexual and reproductive healthcare providers were conducted. Transcribed interviews and observations were imported to Open Code 4.02 for coding, categorizing, and creating themes. Finally, barriers to accessing existing services were explained using thematic analysis.

**Results:**

The study explores contextual barriers to accessing sexual and reproductive health services in five emergent themes. According to providers’ points of view, the barriers include ranging from providers (e.g. poor providers’ competency), health facilities (e.g. supply constraints and unsupportive environment), adolescents (e.g. perceived lack of information and attitude towards SRHs), community (e.g. lack of parental and social support), and broader health system (e.g. poor implementation and multi-sectorial engagement).

**Conclusion:**

As to providers, adolescents face multiple barriers to accessing youth friendly sexual and reproductive health services. Healthcare facilities and all levels of the healthcare system should implement varieties of approaches to increase access to the services for adolescents. Given the lack of progress in utilization of adolescents- youth friendly sexual and reproductive services, the existing strategy should be re-evaluated and new interventions at all levels of the healthcare system are needed. Moreover, implementation research is required at system level factors.

## Background

One in six people worldwide is 10 to 19 years old (adolescents). About 90% of them live in the low- and middle- income countries [1]. In Ethiopia, 24.9% of the population is made from adolescents [1]. Many of them engage in behaviors and experiences that may unknowingly endanger them. Most of the preventable health risk behaviors often develop during adolescence and can progress into adulthood, which may lead to a lifetime of ill health and to death [2, 3]. The risks of avoiding adolescent’s sexual and reproductive health care exposes adolescents to both contextual as well as global health risks including unwanted pregnancy and bad parenthood, difficulties in accessing contraception and safe abortion, and high rates of HIV and other sexually transmitted infections, and sexual violence [3, 4].

Over the past two decades, researchers and health programmers have been implementing youth-friendly model of health services in primary health care setting to address barriers to accessing health care for young people, including adolescents. This is part of the WHO's global call for the development of health services that are relevant to young people worldwide [4, 5]. In general, the establishment of friendly adolescent health services is based on the principles of international human rights treaties, which are based on the principles of equality (including gender equality) and respect for human rights, and protection of human rights. As an important healthcare entity, the Global Adolescent and Youth Sexual and Reproductive Health Service is a framework designed to “safe, effective and affordable” approaches (meeting the personal needs of young people who come to any healthcare facility when they need services and recommend services for friends) [5] to protect young people from many sexually related health problems.

With some additional packages, the services include providing universal access to accurate sexual and reproductive health information; providing safe and affordable contraceptive methods; providing counseling, quality obstetric and antenatal care for all pregnant adolescents; providing safe abortion when unintended pregnancy happens; prevent and manage of sexually transmitted infections including HIV; treat violence against adolescents and manage risky behaviors[6]. As part of the global approach [4, 5], Ethiopia has been striving to improve adolescents and youth health through ratifying national youth policy in 2004 [7]. Following the national youth policy, the country has been developed and implemented two national adolescents and youth reproductive health strategies: the pre-2015 strategy (2007 to 2015) [8] and the post 2015 strategy (2016 to 2020) [9]. This approach is being implemented through both stand- alone modality and integrating sexual and reproductive health services into the basic health services mostly at primary health care settings as well as sometimes at secondary and tertiary level healthcare settings. Although the country has made good progress in designing and implementing adolescent and youth-friendly reproductive health strategy, large number of evidences in almost all corners of the country [9], including the study area [10] showed the use of adolescent sexual and reproductive health services is very low. Studies indicate that the utilization coverage of youth-friendly health services in Ethiopia ranges from 9 to 55% [9] which was among the lowest score of all Sub-Saharan African countries in 2016 [9]. This service utilization coverage even could be likely lower particularly for adolescents because almost all studies cover the age range of 10 to 24 years. Therefore, if one had measured the service utilization among adolescents (10–19 years), the utilization would have been scored low than the above specified range of coverage.

Almost all studies in Ethiopia have reached approximately the same conclusion: low utilization coverage of the services and providing statistical association of some factors with low utilization. Quantitative research on the sexual and reproductive health of young friends in Eastern Ethiopia has shown that service providers' negative attitudes toward serving unmarried adolescents have been linked to the low utilization of the service [11]. Poor interpersonal relationships [12] and many other operational level factors like poor perceived support and poor communication [13, 14] were also barriers to using adolescent and youth friendly sexual and reproductive health care access.

Barriers to using adolescents’ sexual and reproductive health services may be resulted in a synergy of previously studied factors with unexplored contextual factors to the providers’ point of view [14, 15]. Unfortunately, tittle evidence is available about what contextual barriers to accessing adolescent and youth friendly sexual and reproductive health services to the perspectives of healthcare providers. In addition, we believe that a qualitative approach would explore more about the contextual barriers for low utilization than reinventing the wheel (measuring the same thing) using many quantitative approaches. Therefore, the purpose of this study was to explore contextual barriers to accessing the already established youth-friendly services for adolescents in Hadiya Zone, Southern Ethiopia.

## Methods

### Study setting and period

The study was conducted in selected health facilities providing youth friendly sexual and reproductive health services and specialized youth clinic and youth centers in Hossana Town, Hadiya Zone from 20 to 27 February 2020. Hossana city administration is a capital of Hadiya Zone in Southern Ethiopia. There are four government health facilities in the city administration: one referral hospital and three health centers. More than 30 private clinics and 1 private hospital were found in the city administration during the time of data collection. All of the government health facilities and youth centers are expected to provide youth-friendly sexual and reproductive health services according to the national strategy [9]. Accordingly, they provide services through home to home visits by HEWs (Health Extension Workers); population-oriented outreach services delivered by health workers through routine outreach programs; and individual-oriented clinical services that address the sexual healthcare needs of young people including adolescents in all health facilties.

According to department of city sexual and reproductive health services, there were about eight youth centers that have been providing community sexual and reproductive health services in the town before 2015. However, only one youth center is functional during data collection. Private clinics and hospitals are also expected to provide youth-friendly sexual and reproductive health services according to the national strategy. Currently, there are also two known NGOs (Non-Governmental Organizations) clinics that provide youth-friendly sexual and reproductive health services. One functional youth center; three primary health centers; one referral and teaching hospital; one specialized youth clinic; two student clinics; and one city administrative health office that have been providing ASRHs were purposely selected for our study.

### Study design

We used phenomenological study design. We approached the research question from the providers’ perspective regarding barriers to access friendly sexual and reproductive health services. In addition, observation was made by the investigators to supplement experiences of healthcare providers.

### Study participants and sampling strategy

Three primary health care units, one referral hospital, one specialized NGO youth clinic, one city administration health office, one youth center and two educational settings were purposively selected for the study. Criterion sampling was used to select study participants. The criteria used were working at youth-friendly sexual and reproductive health unit of selected healthcare facilities /outreach programs; having better experience of serving at the selected healthcare facilities; being heads of sexual and reproductive health unit of city administration health office; and being coordinator/representative of adolescents at selected youth centers and school clinics/reproductive health clubs. Accordingly, one health professional from the department of sexual and reproductive health services of the city administration; three health professionals serving at youth friendly sexual and reproductive health services; two urban health extension workers who have been working at community level; one coordinator of urban health posts (Kebeles) who has supervising and supporting HEWs; two counselors who have beeen working at youth centers; one high school health and reproductive health club coordinator; and two representatives of college youths who have best experiences of participating in reproductive health clinic/ clubs were selected as key informant interviewees.

### Data collection

In-depth interviews with key informants were used using a semi-structured interview guideline prepared to address barriers to accessing adolescent and youth friendly sexual and reproductive health services. The interviews were completed at a time and place to suit the participants and lasted between 33 and 90 min. Audio recordings were made using a digital recorder and transferred to personal computer for transcription. The recordings were erased from the digital recorder as soon as transferred to password protected personal computers. Collecting data was stopped when key informants fail to provide new information. Memos were used to understand contexts during interview and observation. Observation was made in five health facilities using checklists adapted from guidelines of adolescent friendly services [4, 9, 13] to identify potential barriers to accessing SRHs by adolescents and check general readiness of the health facilities (centers) to provide friendly services for adolescents.

### Operational definition

*Provider* for this study applies to health professionals and non-specialists who were providing youth-friendly sexual and reproductive health services at selected health facilities (health posts, health centers, and hospitals); sexual and reproductive health units of health offices specialized youth clinics; and reproductive health and health related youth centers/clinics in school/college.

*Adolescent/youth-friendly sexual and reproductive health services (AFSRHs/YFSRHs)* for this study apply to the services that are being provided either by integrated or stand-alone modalities through the guiding principles of youth friendliness (accessibility, availability, acceptability, equity, effectiveness and efficiency).

*Adolescents* for this study refer to young people who are in the age range of 10 to 19 years old according to WHO recommendation [4, 6].

### Data management and quality

Prior to data collection, readiness of digital audio recording tool was checked in subsequent interviews. The words of the participants were simultaneously recorded by the investigators to enable for later verbatim interpretation and translation into English. Immediately after completing each in-depth interview, observation of corresponding health facilities was followed through a checklist. Contextual data obtained from interviews and observations were immediately documented in memos to prevent loss on memory during data collection.

### Data processing and analysis

Memos were written immediately after and/or during interviews, observations, and recording ideas as initial analyses. The recorded verbatim audios  were immediately transcribed and translated to English. Again, interview transcripts were re-read line by line and listened to the recordings in order to match a sense of what has been said by each study participant. Phone calls and face to face briefing with study participants were made when some expressions in the audio seem to be confusing while transcriptions were made. Contradicting ideas in subsequent questions were validated during interview by the respondent’s own word and crosschecked with memos. Memos of interviews and observations were crosschecked while transcribing to ensure credibility of data. Transcribed interviews and notes were imported to Open Code 4.02 [16] for coding data, categorizing codes, and creating themes. Initial coding was made from providers’ understanding of the users (adolescents) and model of the service (AFSRHs/YFSRHs); and capturing providers’ experience regarding barriers hindering adolescents from using SRHs. Coding, categorizing (sub-theming) and theming data were carried out by the first investigator. Categorizing and theming procedures were cross-checked by the other investigators and agreed on common categories and themes. We invited an expert to put sample of codes and categories to the emerged corresponding categories and themes for triangulation, respectively. Finally, the phenomenon being studied was explained by emerging categories and themes. Explanations in themes were substantiated by participants’ direct quotations when necessary.

## Results

### Socio-demographic characteristics of participants

A total of 12 providers were participated in the study. The age of participants ranged from 22 to 49 years old with the mean age of 32.5 years old. A health professional from the city’s department of sexual and reproductive health; three health professionals who have been working in the unit of youth-friendly sexual and reproductive health services; a high school reproductive health club coordinator; two urban health extension workers; a coordinator of urban health posts; two counselors who have worked in youth centers; and two adolescent representatives have experience of participating in reproductive health services in student clinics were participated in the study as key informant interviewees (Table 1).

**Table 1:** Socio-demographic characteristics of study participants, Hossana Town, Hadiya Zone, Ethiopia, March 2020 (n = 12)

Characteristics	Number
Sex	
Female	5
Male	7
Age	
20–35	9
36–50	3
Religion	
Protestant	10
Orthodox	1
Catholic	1
Occupation	
Clinical Nurses	4
Public Health Officer	2
Clinical Nurse and Urban Health Extension level IV	2
Psychologist	1
Social Work	1
College Student	2
Educational status	
Degree	8
Diploma(10 + 4)	2
Student	2
Experience in years at AYFSRHs and related centres	
1–9	9
10 or more	3

### Emergent themes and sub-themes

Barriers to using adolescent and youth-friendly sexual and reproductive services were addressed from the points of view of service providers. Five themes explaining barriers to using friendly-sexual and reproductive health services at various levels were identified in the data: provider, health facility, adolescents, community, and health system barriers. Themes were emerged as a result of coding and categorizing participants’ responses to questions addressing barriers that hinder adolescents from using the services. Some of the phrases or sentences of quotes are illustrated from the corresponding codes in each emergent theme and sub-themes (Table 2).

**Table 2:** Themes, sub-themes and sample illustrative phrases or sentences of quotes showing barriers to using AFSRHs, Hossana Town, Sothern Ethiopia, March 2020

Themes	Subthemes	Sample codes	Sample illustrative phrases /sentences of quotes
Provider level barriers	Poor provider competency	Providers lack knowledge	*[…] I was very much confused. I tried to... […]* *[…]... worry about of HIV /AIDS but, for pregnancy."* *[…]If I had understood his feeling, I would have helped him. […]*
		Providers had negative attitude	*[…] I face problem when educating female servants. […]*
		Providers lack communication	*[…]“Are you expecting me to say just I am going to eat it? [..]* *[…]... providers often do not use a direct language. […]*
	Confidentiality breach, disrespect and discrimination of adolescents	Provider breach confidentiality	*[…]... problem of keeping confidentiality of adolescents’ sexual... […]* *[…] I will tell his secrete to his family. […]*
		Providers disrespect adolescents	*[…]... perform abortions without proper counseling and without looking at other options. […]*
		Provider discriminate by sex	*[…]... facilities to seeking help for STI problem. Unfortunately,... […]*
	Providers' lack of follow-up	Providers lack follow-up	*[..]... the service. I referred to but I don’t know their fate […]*
Adolescent level barriers	Fear to violation of confidentiality and cultural taboos	Adolescents fear confidentiality breach	*[…]…are afraid of losing their privacy and confidentiality. […]*
		Adolescents fear providers	*[…] They felt fear and shame. […]*
	Lack of information and attitude towards SRHs	Adolescents lack knowledge	*[…]They don’t worry about the side effects of post pills. […]*
		Adolescents lack attitude	*[..]Many adolescents developed low perceived risk of severity… […]*
	Preference to seeking care and peer influence	Adolescents resist advices	*[…] But, they [adolescents] don’t want to do this [ sexual abstinence].”*
		Adolescents’ decision is influenced by peers	*[…] I see that adolescents are influenced by peers. […]*
	Financial constraints	Adolescents lack money	*[…]... dependent on family income. They lack transportation cost…[…]*
Health facility level barriers	Lack of supply and unsupportive environment	Shortage of supplies	*[…]… many resources and supplies required to provide services. […]*
		No entertainment	*[…] …can’t say that youth friendly sexual and reproductive […]*
		Adolescents lack privacy	*[…]… service itself is not youth friendly at all. […]*
	Inadequate staff and training	Shortage of trained providers	*[…] We intentionally placed the clinic… […]*
		Lack training for providers	*[…]Health extension workers like me should be trained on SRHs. […]*
	Long waiting and inconvenient working time	Long waiting time	*[..] I guess waiting time is very high. […]*
Community level barriers	Community ‘s bad attitude and lack information	Poor community awareness	*[…]Why do employers prevent such adolescents from going to youth health centers?[…]*
		Community negative attitude	*[…]…are considered rude, but those who enter the church are considered polite…[…]*
	Lack of parental and social support	Parents lack discussion	*[…]Parents and teachers don’t talk about sexual and …[…]*
		Church lack discussion	*[…] …settings especially in “Adar program”. […]*
	Inadequate support to schools and youth centers	Teachers lack help	*[…]Reproductive health clubs in the school are not functional. […]*
		Youth centers were closed	*[…]…youth centers which were promoting utilization of SRHs were closed* *[…]*
	Inadequate literacy of sexual health	Lack of curriculum	*"I suggest that curriculum should be designed and educated… […]*
	Presence of unauthorized providers	Unauthorized providers provide contraception service	*[…] They don’t want to go to healthcare facilities for… […]*
Health system level barriers	Poor implementation	Government lack commitment	*[..] Ehh ! I noticed that unemployment is a challenge for not… […]* *[…]But, when such programs phased out; activities of peer… […]*
		Strategy doesn't address adolescents' need	*[..] I think that this strategy has to be revised…[…]*
	Poor multi-sectoral engagement	Lack of multi-sectoral collaboration	*[…]…were providing various sexual and reproductive health services through collaboration.[…]*

### Theme 1: Provider level barriers

Based on this theme, healthcare providers gave personal and collegial experiences that restricted adolescents from accessing SRH services. Under this theme, three sub-themes were emerged: poor provider competency; confidentiality breaches, disrespect and discrimination of adolescents; and lack of provider follow-up.

#### Sub-theme 1.1: Poor provider competency

Under this sub-theme, participants discussed about knowledge, attitude, communication, and technical skill gaps of healthcare providers that may prevent adolescents from using sexual and reproductive health services.

One of the participants working in a specialized youth center noted knowledge gap as:

*“A male adolescent with a special need came to me. I was not familiar with that special need.[…] Instead of having sex with a female, he wanted to orgasm himself. He asked me what problems he would face if he continued to practice’masturbation’. I was very much confused. I tried to advise what I perceived.[…] What I want to underscore here is that we [health care providers] should understand holistic sexual health practice of adolescents. If I fail to help him, he will not revisit the center.” [Female, Age: 20–35, Clinical Nurse].*

Another participant added what his friend had said.

*“My friend is a health professional. He told me that many adolescent girls took injectable contraceptives and went for sex. They [adolescents] don’t worry about of HIV /AIDS but, for pregnancy. Pharmacists inject contraceptives for adolescents of about 13 or 14 years old. Private clinics also do the same thing. […] I don’t know. Don’t healthcare providers know the risk?” [Male, Age: 20–35, Psychologist].*

Urban health extension workers and other participants realized that they did not have enough knowledge to promote and educate about sexual and reproductive health issues for students in schools and adolescents in the community during outreach activities. One of the health extension workers reflected her knowledge and attitude towards sexual health education for adolescents as:

*“[…] I invite other health professionals to provide health education there [school].[…] Parents should pray for their children.[…] I face problem when educating female servants and commercial sex workers[..]” [Female, Age: 20–35, Health Extension Worker].*

Nine out of 12 participants felt that the communication between providers and adolescents was influenced by a number of factors including cultural factors. The majority of the participants felt that adolescents did not talk openly to providers about sexual health problems for various reasons. This in turn prevented them from using SRH services. One of the participants said:

*“I saw a 10-years-old boy. He came to our [center] condom outlet box. I was expecting as if he was going to a game center. But, he picked up a packet of condoms and kept in his pocket. I was shocked and asked him why he did that. He replied to me’Are you expecting me to say just I am going to eat it?. If I had understood his feeling, I would have helped him in the first place. That was my problem.” [Female, Age: 20–35, Clinical Nurse].*

Another more experienced participant sated communication barriers as:

*“Adolescents want everything to be clear about sexual health in their mood. They would like the media to speak openly. We [health professionals] need to talk openly with adolescents. However, we often do not use a direct language. We don’t counsel adolescents with joking and loving. For example, most professionals use ‘sexual intercourse’ [Amharic: Yegibre Siga Gingnunet] while counseling adolescents. What does it mean for adolescents?.” [Female, Age:36–50, Clinical Nurse].*

The researchers’ observation also confirmed that other health workers either hadn’t knowledge or positive attitude towards youth-friendly services. During the interview, the investigators observed that other health care providers were disturbing both counselors and adolescents; moving in and out for their own purposes. Such phenomenon was common in almost all health facilities, with the exception of one specialized youth sexual and reproductive health center.

#### Sub-theme 1.2: Confidentiality breach, disrespect and discrimination of adolescents

This sub-theme discusses the experience of participants and their colleagues regarding breaching confidentiality as barriers to accessing sexual and reproductive health services. The sub-theme also explains disrespectful and discriminant behavior of healthcare providers.

One of the participants witnessed her own mistake how she was breaching confidentiality as:

*“If a 15 year- old boy wants to have sexual partner, I will tell his secrete to his family. Because, I see that adolescents are influenced by peers not by their own decision.” [Female, Age: 20–35, Health Extension Worker].*

A counselor in the selected youth center also witnessed how healthcare providers disclose adolescents’ secrete as.

*“Healthcare providers have a problem of keeping secrets about adolescents’ sexual issues. Let me give you an example that makes me angry. A girl from a school was attending phase-1 training on HIV / AIDS. She told a secret to the trainer [a male health care provider]. She told him that her families scheduled a female genital mutilation ceremony. Instead of counseling and linking her with the right service, he disclosed her secrets to phase- II trainees. One of the attendees asked me who had been sent using key identifiers told by the trainer. I called for a school head to know who she was. He told me her name. […] I don't know if it should be made public. I think this is a problem.” [Male, Age:20–35, Psychologist].*

One participant reflected discrimination made by a healthcare provider based on sex of adolescent as:

*“Approximately, 17 or 18 years-old male adolescent told me a hurting story. He went to health facilities to seeking help for STI problem. Unfortunately, the healthcare provider was a female. She told him that he couldn’t get the service he wanted because a male provider was not around. So, she let him go to another private clinic. This teenager and many others may not want to revisit such healthcare services. […] Many healthcare providers believed that condom demonstration for male adolescents should be done by a male healthcare provider.[…]” [Female, Age: 20–35, Clinical Nurse].*

One participant also shared his experience as sign of disrespecting adolescents.

*“I have often noticed that private health care providers work more for money than providing adolescent friendly services. I heard that they inject Depo [contraceptive] for girls aged 14 and 15 without asking why those girls came to the clinic. In addition, most private health care providers perform abortions without proper counseling and without looking at other options.” [Male, Age:20–35, Psychologist].*

### Sub-theme 1.3: Providers' lack of follow- up

This sub-theme explains providers' lack of follow-up as a barrier to accessing sexual and reproductive health services. Majority of the health care providers reported that they had not followed up adolescents once they had provided services.

One of the participants described the follow- up problem as:

*“[…] Both of them were students between 15 and 16 years old. They came for abortion service, but the service was not available in our center. I refer them to a private clinic. I guess they didn't have money. I referred to, but I didn’t know their fate. I didn't know where they went for such service.” [Female, Age: 20–35, Clinical Nurse].*

A coordinator in a youth center also said that trained peers did not monitor other peers..

*“We [Youth Association Center] trained peers. Each peer is expected to network up to 10 teenagers. They graduate every three years and other new groups continue to be trained. We have streamlined the process. Accordingly, peers perform their duty anywhere outside the center. But, we don’t have strong follow up system.” [Male, Age: 20–35, Psychologist].*

One participant from the city's department of sexual and reproductive health services stated the follow- up gaps as:

*“Previously, we [City Administration Health Office] have provided supplies and equipment (educational materials, musical instruments, loudspeakers and other necessary equipment) to the youth center. These devices were stolen. I don't know how. We asked Office of Women, Children and Youth (OWCY) though official letter to explain how it happened and how it could be prevented. Now, this youth center stopped its function. There are other youth centers that have stopped working for similar reasons. But, we found nothing.”* **[Male, Age: 36–50, Clinical Nurse].**

### Theme 2: Adolescent level barriers

This theme was emerged to explain the experience of healthcare providers and their colleagues regarding barriers that prevented adolescents from using friendly sexual and reproductive health services. Four sub-themes were emerged: fear to violation of confidentiality and cultural taboos, lack of information and poor attitude towards SRHs, preference to seeking care and peer influence, and financial problems.

#### Sub-theme 2.1: Fear to violation of confidentiality and cultural taboos

Under this sub-theme, major misunderstandings, legitimate breaches and fears due to cultural taboos were explored. Most of the participants reported that adolescents perceived violation of confidential information by the healthcare providers. In addition, adolescents were also afraid of being seen by other people during their visit to youth friendly services. One of the participants gave an example of how adolescents were prevented from accessing nearby sexual and reproductive health care services.

*“[…] Overcrowding in the majority of our public health centers is not safe for adolescents. As a result, adolescents are afraid of losing their privacy and confidentiality. For example, I know some pregnant adolescents who went for abortions up to 32 km to hide it.” [Male, Age:20–35, Public Health Officer].*

Another participant added:

*“[…] A pregnant adolescent and her sexual mate were not interested to be referred to government hospital for abortion. They felt fear and shame. They perceived as if they are not accepted by health care providers working in that government hospital." [Female, Age: 20–35, Clinical Nurse].*

#### Sub-theme 2.2: Lack of information and attitude towards SRHs

This theme explores the experience of providers how adolescents’ poor knowledge and attitude towards SRHs prevented them from accessing the service. Accordingly, ten participants reported information gap of adolescents regarding sexual health and services ranging from not knowing where to go for seeking help to developing negative attitude towards the service.

One of the participants exemplified adolescents’ misconceptions and poor practice regarding contraception.

*“Many teenagers often take post pills every morning at our clinic. […] I provide it to them. You know, they can get it from pharmacies if I refuse to do so. They have no access problem. They don’t worry about the side effects of post pills. Many of them fear pregnancy more than any other health risk. They do not pay attention to HIV / AIDS and other STIs. I do not know. Either they do not understand or there is a fear of pregnancy.” [Female, Age: 20–35, Clinical Nurse].*

Participants gave a testimony that many adolescents had developed negative attitude towards sexual health services. One of the participants stated the problem as:

“*Many adolescents developed low perceived risk of severity about sexually transmitted infections including HIV/AIDS. For example, I have heard many adolescents who have wrong perception. They [adolescents] reflect the incurable HIV/AIDS as if it was curable diseases like other STIs. […] It seems that they don’t want to listen to any counselor at all. Female adolescents in schools act like commercial sex workers. I can say that paradigm of sex commercialization is seemed to shift from poor women to students [adolescent].” [Male, Age: 20–35, Psychologist].*

#### Sub-theme 2.3: Preference to seeking care and peer influence

This sub-theme explores providers’ experience on how choice of care and influence of peers affect decision to use SRHs. Providers reported that adolescents seem to have concern on age and gender of healthcare providers to seek help from health providers. Healthcare providers had considerable dialogues about whether gender-matched providers were most appropriate.

*“I had experienced that many adolescents seek providers of similar sex. They perceived that a male adolescent who seeks help for STI [sexually transmitted infection] problem should be helped by a male care provider. […] It was for females too. I also prefer to male if I were male.” [Female, Age: 20–35, Clinical Nurse].*

Providers also reported that adolescents seemed to care about the age of healthcare providers to access sexual and reproductive health services. One participant stated adolescents’ preference as:

*“[…] If they [adolescents] missed me [older health care providers]; they try to search for other two female professionals who were working here [Youth Friendly Service Clinic] for many years. The current problem is that adolescents don’t want to be served by adolescent health professionals. They feel like to be served by elders. I don’t know.”[Female, Age: 36–50, Clinical Nurse].*

Half of the participants had mentioned that adolescents were influenced by their peers in decision making of accessing SRHs. Majority of the healthcare providers reported that peers had provided health information and had promoted where and when youth friendly services had been provided.

#### Sub-theme 2.4: Financial constraints

More than half of the participants believed that joblessness and limited access to household resources hindered many adolescents from accessing SRHs due to cost of service delivery, supplies, and transportation.

One of the interviewees described financial problems as:

*“Yea, many adolescents lived separately from their families for reasons such as education. They are dependent on family income. They lack transportation cost to come to the health facility for SRHs. They can’t afford lunch or tea cost while spending for a day.” [Male, Age: 36–50, Public Health Officer].*

Another participant added:

*“[…]They [housemaids] have a right equivalently as other family members as possible. Maids are often considered diseased in fact they are considered to have been sexually abused by a family member. […] I have met many female maids who have been sexually abused by family members like male adolescents, household heads, neighbors and others. […]When maids get pregnant, employer(s) let them leave their home. Employer(s) don’t want to listen to problem of household maids. You know, such maids may not have money to access any sexual health services.” [Male, Age: 20–35, Psychologist].*

### Theme 3: Health facility level barriers

This theme focuses on the experiences of participants and their colleagues concerning barriers for adolescents from using existing services. Three sub-themes were emerged: lack of supply and unsupportive environment, long waiting and inconvenient working time, and inadequate staff and training.

#### Sub-theme 3.1: Lack of supply and unsupportive environment

The sub-theme discusses providers’ experience regarding unavailability/limitation of resources needed to perform key activities to meet the sexual and reproductive health needs of adolescents. On the other hand, unsupportive environment to responding to the needs of adolescents has been explored. The majority of participants strongly pointed out unavailability of supplies required to provide adequate and appropriate services for adolescents. Lack of written guidelines and lack of educational materials such as posters and flyers were also reported. One of the participants explained the problem as:

*“I counsel how to use contraceptives. Previously, all packages were here [Youth clinic]. In this unit, supplies were fully supported by IFPH [Non-governmental organization]. But now, there is no support. […] After IFHP phased out, there is a shortage of many resources and supplies required to provide services. We [health professionals] sometimes do not have some long-term contraceptive methods, HCG [pregnancy test] and STI medicines.” [Female, Age: 36–50, Clinical nurse].*

Providers cited inadequate physical space and privacy as institution-level barriers to using adolescent sexual health services. The service providers also pointed out that youth clinics did not have enough entertainment and spaces. The researchers’ observation during the interview also proved that almost all YFS (youth-friendly service) clinics have only a single unit and is not separated from adult outpatient units. Some centers were also close to HIV/AIDS clinics.

Most participants agreed that the lack of privacy in health facilities and hospitals has resulted in fear of being seen by friends, relatives, or other community members. One of the participants described the situation as:

*“[…]An auditor and a counselor share a similar room. An auditor gets out of the room when clients come and comeback when clients leave a room. In this case, the service itself is not youth-friendly at all. I think this is unsafe even for adults. Adolescents need private and confidential services. Adolescents fear others and feel shame if they are seen by others. They don’t revisit such centers.” [Male, Age: 36–50, Clinical nurse].*

#### Sub-theme 3.2: Inadequate staff and training

Providers felt that inadequate training on adolescent sexual and reproductive health was one of the barriers to providing quality sexual health services. In addition, inadequate number of trained staffs was also mentioned by the majority of the participants as a barrier to providing quality services. Participants described how they were facing challenges of providing services related to HIV/AIDS due to lack of technical updates. One of the participants said:

*“[…] Health extension workers like me should be trained on sexual and reproductive health services. […] I took it as part of my course when I was in college. I mean, 9 years ago. I also suggest that teachers and health development armies should be trained.” [Female, Age: 20–35, Health Extension Worker].*

Another participant described a shortage of healthcare providers as:

“*We [Hospital Youth Friendly Clinic] have many clients. I mean, flow of client is very high. We intentionally placed the clinic [Youth Friendly Clinic] with other specialty outpatient clinics. What I mean is that our trained healthcare providers give other healthcare services.” [Male, Age: 20–35, Public Health Officer].*

#### Sub-theme 3.3: Long waiting and inconvenient working time

This sub-theme explores the experience of providers and their colleagues about what hinders adolescents from accessing services. Inconvenient working time and long waiting time were frequently mentioned barriers to accessing services by adolescents. One of the participants said:

“*I guess waiting time is very high. We [Hospital youth friendly service providers] receive many clients from the town, and rural Kebeles of the Zone and other Zones.[…] We [healthcare providers] can’t address majority of the adolescents coming to our center. We focus on adolescents who have special sexual and other health problems due to overburden. I usually observe that they don’t want such clinic because it is overcrowded.” [Male, Age: 36–50, Public Health Officer].*

### Theme 4: Community level barriers

This theme explores the experience of providers alongside the community that prevents adolescents from accessing existing sexual and reproductive health services. Under this theme, five sub-themes were emerged: community’s bad attitude and lack of information; lack of parental and social support; inadequate support to schools and youth centers; inadequate literacy of sexual health; and presence of unauthorized providers.

#### Sub-theme 4.1: Community’s bad attitude and lack information

This sub-theme explains providers’ points of view about community’s knowledge and attitude towards sexual health services that hindered adolescents from accessing the service. Almost all participants agreed that community’s negative attitude towards sexual health issues in one way or another has negatively affected adolescents from using the service. One of the participants stated the perception and attitude of the community as:

*“Adolescents who come to our [Youth Clinic] centers are considered rude, but those who enter the church are considered polite as perceived by church persons. Some church fathers perceived that anyone can be cured of any disease, such as HIV / AIDS. […] Therefore, they do not go to health care providers. Church fathers do not discuss sexual matters.” [Male, Age: 20–35, Psychologist].*

Another participant stated that the community would level adolescents as "bad" boys or girls if  they went to youth clinics.

*“[…] Guess what could have been male adolescents faced if any of the family members had got ‘condom’ in his pocket. A similar problem would happen if female adolescent was found to have a sexual couple. Parents or community see children as if they were guilty if they were found to go to health facilities for sexual health issues.”* [Female, Age: 36–50, Clinical Nurse].

One participant explained how communities had violated adolescents’ right  of using sexual and reproductive health services.

*“[…] I see that many investors employ early adolescents (approximately below 15 years of age). I mean, they are too kid. The household owners did the same. Let it be. Why do employers prevent such adolescents from going to youth health centers? Employers need to know that all adolescents have the right to use and complain about their sexual and reproductive health services regardless of their economic, social, and sexual status.” [Female, Age: 36–50, Clinical Nurse].*

#### Sub-theme 4.2: Lack of parental and social support

This sub-theme explores the experience of providers whether parents and other community members support for adolescents to use SRH services. Punishing, discriminating, and controlling decision making of adolescents for varieties of reasons were mentioned by the majority of participants that indicate the lack of parental and/or social support. In addition, eleven in twelve participants indicated that parents lacked discussion with their children about sexual and reproductive health matters. One of the participants clarified the idea as:

*“[…] Parents and teachers don’t talk about sexual and reproductive health issues. […] When I was in elementary school, my mother used to teach me about sexual health like contraception use and other issues. She used to tell me by relating with spiritual issues. […] She accepts when I want to go health facilities for help. I thank God for having such mother. I didn’t face any problem. But, mine is not common to all parents." [Male, Age: 20–35, Student representative of RH and HIV/AIDS club].*

Another participant stated that lack of discussion with religious people prohibited adolescents from using sexual and reproductive health services.

*“[…] Adolescents also practice sexual contact in religious settings especially in night program [“Amharic: ‘Adar’]. Majority of the church fathers and followers don’t want to educate adolescents about sexual and reproductive health issues. Religious fathers have to work on more about sexual education for adolescents at early age. They have to promote use of sexual and reproductive health services when adolescent need to use such services.” [Female, Age: 20–35, Clinical Nurse].*

#### Sub-theme 4.3: Inadequate support to schools and youth centers

Under this sub-theme, majority of interviewees pointed out many barriers that may prevent adolescents from using existing services because teachers, community and other stakeholders had not sufficiently been supported by the health facilities and the health system. One of the participants clarified the situation as:

*“Our [Youth centers] link with health facilities had broken down. Almost all of youth centers which were promoting utilization of SRHs were closed. Currently, only one out of ten centers in the town is functional. Members of the centers had left due to various reasons.[…] Medias ignored talking about HIV/AIDS and other SRH related problems. […] Majority of reproductive health clubs in the school are not functional. […] Church fathers seem to let the community not to worry about HIV/AIDS.”[Male, Age: 20–35, Social Worker].*

#### Sub-theme 4.4: Inadequate literacy of sexual health

This sub-theme focuses on the perspectives of health care providers regarding inadequacy of sexual literacy as barriers to accessing ASRHs. Half of the participants believed that absence of formal sexual health education in schools could also be a barrier to accessing ASRHs for adolescents.

One of the participants explained how the absence of formal sexual health education affected adolescents regarding SRHs utilization.

*“I suggest that age appropriate sexual and reproductive health course should be given starting in primary schools. […] I remember my biology teacher when I was a student. He [teacher] called different parts of our body, but silent when he reached at our reproductive region. But, we know everything, even if teachers don't tell us. That would make students shy or fear to go to health facilities for help.” [Male, Age: 20–35, Psychologist].*

One of the participants added the need of formal sexual health education in schools.

*“I suggest that curriculum should be designed. Adolescents should be educated starting from elementary school about sexual health. […] Let me ask you, why SRH services like condom was not available in high schools while it was available in Universities? Is it because of that teachers are afraid of encouraging sex between students? That is wrong. Because adolescents know everything about sex though we don't tell them about.” [Female, Age: 20–36, Coordinator of RH and HIV/AIDS club in a school].*

#### Sub-theme 4.5: Presence of unauthorized providers

This sub-theme mainly focuses on participants’ experience whether the presence of unauthorized providers led adolescents not to use ASRHs. Five participants mentioned that adolescents used unapproved providers instead of accessing services from accredited health care facilities. One of the participants said:

*“[…]They[adolescents] access contraceptives from private pharmacies. They don’t want to go to healthcare facilities for counseling. Currently, majority of adolescents want to use contraceptive pills than using other methods like condoms. I remember when I was working with DKT Ethiopia. Previously, DKT was selling 10 Ethiopian Birr whereas pharmacies were selling 15 Ethiopian Birr. But, currently, DKT is selling 30 Ethiopian birr whereas pharmacies are selling 150 Ethiopian Birr.” [Male, Age: 20–35, Psychologist].*

Another participant also said:

*“Currently, adolescents are not using contraceptives. I think they might use contraceptives outside of this center. But, they come when problems related to pregnancy and sexually transmitted infections happen. As you know, they can access contraceptives everywhere (pharmacies). Adolescents come with complaints of menstrual irregularities. I guess; this is because they took emergency pills repeatedly. [Female, Age: 36–50, Clinical Nurse].*

### Theme 5: Health system level barriers

This theme explores experience of providers regarding a broader health care system that could negatively affect the use of services by adolescents. Two sub-themes were emerged: poor implementation and commitment, and low stakeholder engagement.

#### Sub-theme 5.1: Poor implementation and commitment

Providers described many health system level barriers that had prevented adolescents from accessing sexual and reproductive health services. Barriers mentioned by the participants in the study area included lack of funding, fail to job creation, and lack of attention to youth friendly-services.

One of the participants described unemployment as a barrier to accessing ASRHs as:

*“Ehh! I have noticed that unemployment is a challenge in terms of access to health care, including sexual health services. […] One day, a girl asked me to carry my bag because she had no job. There is no job creation here. Do you think adolescents accept me when I talk about sexual health services while there are many competing needs?” [Female, Age: 20–35, Health Extension Worker].*

Majority of the participants also indicated poor commitment of the government. One of the participants explained how the government lacked commitment as:

*“[…] AFSRHs was more effectively done when we were supported by non-government organization like pathfinders [NGO] and IFPH [NGO]. […] During that time, peers were calling for their friends who face difficulties in sexual health problems in the community and link to our center [Youth Clinic].Peers also provide health information and promote where and when the youth friendly services were being provided. This was made through payment to selected peers for the work they did. But, when such programs phased out, activities of peer becomes down.[…] We [health facilities] couldn’t make it sustainable." [Male, Age: 36–50, Public Health Officer].*

Two of providers complained that implementation of the national strategy of youth friendly sexual and reproductive health strategy was not adequately responding to the sexual health need of adolescents. One of the providers complained his doubt whether the strategy brought changes to adolescents’ health seeking behavior as:

*“Yea, we have a strategy on youth- friendly sexual and reproductive health services. But, I don’t think that it had brought intended results. How it was being implemented? What were the gaps? […] Is it really addressing sexual and reproductive health needs of adolescents? I think that the strategy has to be revised and include the current needs of adolescents.” [Male, Age: 36–50, Clinical Nurse].*

#### Sub-theme 5.2: Poor multi-sectoral engagement

The sub-theme discuses opinion of participants regarding the lack of cooperation among stakeholders in addressing sexual and reproductive health needs of adolescents. Four participants complained the lack of cooperation among health professionals, health facilities, schools, youth centers, adolescents and youth-oriented sectors, and various governmental and non-governmental organizations. One of the participants described the problem as:

*“Okay, as you know community health activities increase demand in using any health care services including SRHs. Previously, we [Staffs of youth centers] were providing various sexual and reproductive health services through collaboration. We were supported by various NGOs. But, currently these activities are not sustainably being implemented. […] Communities don’t own or support such centers [Youth Centers]. […] Government does not support the centers. Why governments consider youth centers as legal institutions?” [Male, Age: 36–50, Psychologist].*

## Discussion

Despite many years had been counted in implementing a youth-friendly model of sexual and reproductive health services, many quantitative studies have shown low utilization scores among young people including adolescents in Ethiopia [9]. The services have been provided either through integrating into basic health services in the healthcare facilities or stand-alone modalities sometimes. This model of healthcare for adolescents might be ineffectively delivered so that unable to reach adolescents. Therefore, this study explored the perspectives of service providers regarding barriers to accessing adolescent sexual and reproductive health services.

The study has identified modifiable healthcare access barriers why adolescents had not used the services in five organized themes: providers, health facility, adolescents, community, and health system level (Table2). Barriers in each theme may at least affect one of the basic dimensions (accessibility, availability, acceptability, equity, effectiveness, and efficiency) of the youth -friendly model of care [4, 14].

The study found that all providers outside health care facilities (health centers, youth centers, and school and college clinics/SRH clubs) and some health care providers felt that they knew how and what to do on sexual health services for adolescents. Although approaches vary, studies in Ethiopia [17], Ghana [18], South Kenya [19], India [20], and Vanuatu, the South Pacific Islands [21] show that such factors affect health care providers and in turn affect adolescents.

We expect urban health extension workers to serve the urban community where literacy is higher than the rural community in Ethiopia. Hence, Health Extension Workers (HEWs) need to have better technical updates/qualification in SRH packages to gain acceptance in the community. However, the findings of this study -show that health extension workers even could not accurately identify adolescents and what services were appropriate for adolescents. Similarly, many participants reported having difficulty in communicating with adolescents while providing AFSRH services during the visit. The findings agree with other research conducted in primary care settings in South Africa [15] which found the relationship between providers and clients was limited to brief instructions and cursory explanations.

Our study explored the confidentiality and discrimination of adolescents by demographic backgrounds like sex and age. This may affect adolescents’ choice of care (autonomy) and distance them from accessing the existing friendly sexual and reproductive health services. The findings of this study agree with studies conducted in the South Pacific Islands [21] and Bolivia [22] that sex and age were important factors to consider while reaching adolescents. Providers should closely follow-up adolescents regarding outcomes of the services such as changes in behavior after they provide services as shown by the WHO [23]. In addition, the healthcare system and key stakeholders need to work in collaboration with a healthcare provider to increase health- seeking behaviors of adolescents in the country. Unfortunately, as to the participants’ witness in our study, almost all approaches were not effectively considered for the contexts as intended to reach adolescents for better SRH service utilization.

Adolescent side barriers like fear of confidentiality breach, cultural taboos, financial constraints, unmatched choice of care, peer influence, lack of information, and poor attitude towards SRHs were important challenges affecting service utilization. Awkwardly, the majority of providers in the study were mentioning the above challenges as limiting factors for utilization of the service. The findings of our study were also congruent with studies done in Ethiopia [24], Ghana [25], and Nepal [26]. In addition, according to our findings, adolescents wanted to be served by providers of older age and the same sex. According to WHO [23], these findings seem to have an inverse association.

Many of the participants in this study repeatedly complained shortage of supplies such as modern contraception methods, essential medicines, pregnancy, and STI test kits at youth clinics. Participants also criticized the service environment for its unfriendliness to adolescents to use the services more easily and equitably. Furthermore, the majority of participants identified the inconvenient work schedule, the inadequacy of trained staff, and limited training for providers. All the above challenges negatively impact principles of adolescent-friendly services that may lead to ineffective delivery of services. Although the circumstances somewhat vary, our findings are consistent with the studies conducted in Ethiopia [24] and Tanzania [27, 28].

Under the theme of community- level barriers, participants raised many perceived and/or actual barriers to accessing sexual and reproductive health services. Although identical studies to our study are difficult toget, this finding seems to be consistent with other findings in Ethiopia [24] and Rwanda [24],and Ghana [25]. Our findings imply that community sexual and reproductive health promotion is required to support adolescent-friendly services among the general population, teachers, religious followers, and other stakeholders. Creating supportive environment for adolescents at all levels of the broader health system to deliver an effective friendly SRH service model of care for the study area and the country at large [29]. Creating a supportive environement for adollescents is really contextual and needs involvement of multiple sectors, professionls, and other stakeholders that needs system level thinking [29]. 

Providers in the study repeatedly complained lack of discussion between adolescents and parents. The findings of this study agree with other studies in India [20], Kenya [30] and Nigeria [31], and Ethiopia [32, 33]. In addition, studies also showed that lack of parental knowledge and positive control [32], gender-selective influence [34], and poor parental involvement in planning SRHs [35, 36] are barriers to accessing AFSRH services. Our findings also suggest the need to revise the current strategy to include and collaboratively work with parents, teachers, and religious persons at the community level. This should be started at the early age of adolescents to help adolescents plan their sexual needs and use health services without fear and shame.

In our study, the majority of participants had perceived that poor sexual health literacy at and earlier age could be a barrier to knowing, planning, and accessing sexual and reproductive health services. Fear of encouraging earlier sexual activity [37] is one of the most cited reasons for not providing sexual education in formal settings. But, stop talking about sex and sexuality can’t delay sexual initiation [35, 37, 38] because adolescents know everything about it in the age of health information technology. Instead, sexuality education or literacy may create a sexually healthy future generation and fulfills sexual health for all [35, 37, 38]. Therefore, we suggest schooling about sex, sexuality, and sexual health services through designing age-appropriate, context-based and need- based curriculums starting from medium cycle [above grade 5] education. Besides, outreach programs brought limited impact on healthy sexual health behaviors and practices among young people in Sub-Saharan Africa including Ethiopia [36, 38], and hence should be done at school.

Our study showed that unauthorized providers were seriously accused of prohibiting adolescents from using friendly sexual and reproductive health services. This finding is consistent with the study conducted in Addis Ababa that found illegal providers like Pharmacies abundantly provide emergency contraception for teenagers [39]. This implies that the need to establish need-based SRH provision systems like establishing youth clinics in high schools and strengthening youth centers to have healthcare providers with sufficient supplies to make the service closer to adolescents. Broader system-level challenges also call for urgent actions through multi-sectoral collaboration to increase access to sexual and reproductive health services in the study area.

Although the revised national strategy of the country included service delivery modalities(integrated or stand-alone ASRHs in the house to house, outreach programs,and individual clinics settings) [9], the need for trained staff, community link, parental and community support, developing need-based sexual education curriculums, working with private ASRHs providers, technological and material supplies and usage should further be included in the service standards and packages of the coming revised national adolescent strategies.

## Conclusion

As to providers, adolescents face multiple barriers to accessing youth- friendly sexual and reproductive health services. Barriers exist at the levels of providers, health facilities, adolescents, community, and broader health system. Because of the complexity of barriers hindering adolescents from using friendly sexual and reproductive health services in primary care settings, multiple adolescent oriented approaches should be implemented. Given the lack of progress in the utilization of adolescents-friendly sexual and reproductive services, the existing strategy should be re-evaluated and new interventions at all levels of the healthcare system are needed. The findings of this study in each theme could also help the zonal health office to review its adolescent health services at health facilities and improve mainly the availability of adolescent health services. The issues of capacity building; uninterrupted SRH commodities and supplies; and partnership should be given priority in addressing SRH services to make the service available. Moreover, implementation research is required at all levels of the health system in the country.

## Data Availability

All the data are available in the manuscript. Supplementary files may be provided through reasonable request.

## Abbreviations

RH: Reproductive health
SRH: Sexual and reproductive health
AFSRHS: Adolescent-friendly sexual and reproductive health services
YFS: Youth-friendly services
NGO: Non-governmental organization
IFHP: International family health program
STI: Sexually transmitted infection
HIV/AIDS: Human immunodeficiency virus/acquired immune deficiency syndrome
